# Multidisciplinary management of adolescents and young adults (AYA) sarcoma: A successful effort of an adult high‐volume cancer center

**DOI:** 10.1002/cam4.6289

**Published:** 2023-06-27

**Authors:** Alexia Francesca Bertuzzi, Maria Susanna Grimaudo, Alice Laffi, Laura Giordano, Nicolò Gennaro, Umberto Cariboni, Licia Vanessa Siracusano, Vittorio Quagliuolo, Piergiuseppe Colombo, D’Orazio Federico, Salvatore Lorenzo Renne, Cristina Specchia, Ferdinando Cananzi, Andrea Marrari, Pierina Navarria, Primo Andrea Daolio, Stefano Bastoni, Armando Santoro

**Affiliations:** ^1^ Department of Oncology & Hematology IRCCS Humanitas Research Hospital Rozzano Italy; ^2^ Department of Biomedical Sciences Humanitas University Italy; ^3^ Biostatistic Unit IRCCS Humanitas Research Hospital Rozzano Italy; ^4^ Feinberg School of Medicine Northwestern University Chicago USA; ^5^ Department of Surgery IRCCS Humanitas Research Hospital Rozzano Italy; ^6^ Department of Pathology IRCCS Humanitas Research Hospital Rozzano Italy; ^7^ Department of Radiology IRCCS Humanitas Research Hospital Rozzano Italy; ^8^ Department of Gynecology IRCCS Humanitas Research Hospital Rozzano Italy; ^9^ Department of Oncology Istituto Ortopedico Rizzoli Bologna Italy; ^10^ Department of Radiotherapy and Radiosurgery IRCCS Humanitas Research Hospital Rozzano Italy; ^11^ Department of Orthopedics ASST‐Gaetano Pini‐CTO Milano Italy

**Keywords:** adolescent, AYA, oncology, sarcoma, young adult

## Abstract

**Introduction:**

The aim of this retrospective study was to investigate the clinicopathological characteristics of AYA sarcomas and their clinical outcomes at a high‐volume single center.

**Methods:**

Demographic, clinicopathological data on the diagnosis, treatment and follow‐up of all sarcoma patients aged 16–39 years (ys) observed at our Institute between January 2010 and December 2021 were retrospectively collected, including diagnostic (TTD) and treatment delay(TTT), clinical outcomes (OS and PFS), and late‐treatment effects.

**Results:**

We identified 228 AYA patients, median age 30 years, 29% ≤ 25 years, 57% males, 88% soft tissue sarcomas (STS), and 12% bone sarcomas (BS). Among STSs, 13% were small round cell tumors (SRCT), 52% intermediate–high‐grade, 24% low‐grade STSs. Among BS, 32% were high‐grade. Median TTD and TTT were 120 (0–8255) and 7 days (0–83), respectively. Surgery was performed in 83%, radiotherapy in 29%, and systemic therapy in 27%. Median follow‐up was 72.9 months(1.6–145), 5‐year and 10‐year OS were 78.5% and 62%, respectively. Kaplan–Meyer analysis showed a significantly better 5‐year OS and PFS for patients with >92 days of TTD (OS 85.7% vs. 66.7%, *p* = 0.001, PFS 50.2% vs. 24.9%, *p* = 0.009). According to age (≤25 years vs. > 25 years), 5‐year OS was 69.8% versus 82.2%, respectively (*p* = 0.047).

**Conclusion:**

Our analysis confirmed previous data on sarcoma AYA patients followed in a referral center. Unexpectedly, diagnostic delay was not associated with poor OS and PFS. Patients <25 years showed a poorer prognosis due to the higher incidence of SRCT.

## 1 INTRODUCTION

Sarcomas are rare cancers of mesenchymal origin that arise either from soft tissues and bones, accounting for <1% of malignant tumors in adults. Conversely, they represent almost 8% of all cancers in adolescents and young adults (AYA) patients, worldwide identified as the population between 16 and 39 years of age. In this unique group of patients, the clinical outcome in soft tissue sarcoma (STS) has been reported as more favorable compared with the older population. Conversely, typical pediatric histotypes (i.e., small round cell tumors, SRCT) seem to show a worse prognosis in AYA patients compared with younger patients, likely due to a peculiar tumor biology and poorer tolerability of the chemotherapeutic regimens.^1^, ^2^, ^3^, ^4^ This prognostic gap might also be justified by the hypothesized diagnostic delay, often leading to diagnosis in advanced stage, and a limited enrollment in clinical trials.^4^, ^5^, ^6^, ^7^, ^8^ A proper comprehension of the AYA scenario, known as a no‐man's land standing between pediatric and adult oncology, has required a global multidisciplinary effort and an exciting challenge for oncology health care professionals. In this field, among subtypes of cancers, sarcoma may represent the most appropriate model of care in AYA oncology.^9^, ^10^ Indeed, both sarcomas and AYA cancers are rare tumors that require a multidisciplinary diagnostic and therapeutic approach to improve outcomes and reduce long‐term treatment‐related sequelae. Caring for AYA patients requires dedicated time and resources to promptly assist patients in a comprehensive framework, including a tailored focus on their medical and psychosocial issues throughout their cancer journey.^11^, ^12^, ^13^, ^14^ Although already running even before 2018, a specific AYA Program was then officialized within our tertiary cancer center to meet the unique AYA needs, which may be missed or not prioritized due to the competing demands of a high‐volume Adult Oncology Department.^15^ In this retrospective analysis, as a reference national center for the diagnosis and treatment of sarcoma and AYA oncology, we report our clinical experience in this peculiar cohort of patients.

## 2 MATERIALS AND METHODS

### 2.1 Patients data

We retrospectively reviewed the electronic medical records of all sarcoma patients aged 16–39 years (ys) treated at the Humanitas Research Hospital between January 2010 and December 2021.

Information extracted from each individual's medical records included demographic data, date of diagnosis and onset symptoms, time to diagnosis (TTD, the time from the symptoms onset and pathological diagnosis), sarcoma histotype, the primary site, the status of disease at diagnosis (localized, locally advanced, and metastatic disease), the treatment strategies (surgery, radiotherapy, chemotherapy, and targeted therapy), the time to treatment (TTT, the time from visit to the disease management), relapse/progression data, and survival status. In addition, we reported on the long‐term treatment toxicity, including cardiac issues, functional impairment, and the onset of secondary tumors.

The local IRB committee obtained an authorization waiver prior to carrying out the present analysis.

### 2.2 Statistical analysis

Descriptive statistics were used to analyze the study population. Patients' characteristics have been summarized as numbers and percentages or as medians and range. The Kaplan–Meier method and the Cox proportional hazard model have been used for survival estimates, considering a *p* value ≤ 0.050 as statistically significant. Relapse analyses used the date of data collection as the censorship date. All analyses have been performed using SAS software (version 9.4, SAS Institute Inc., Cary, USA).

## 3 RESULTS

**FIGURE 1:**
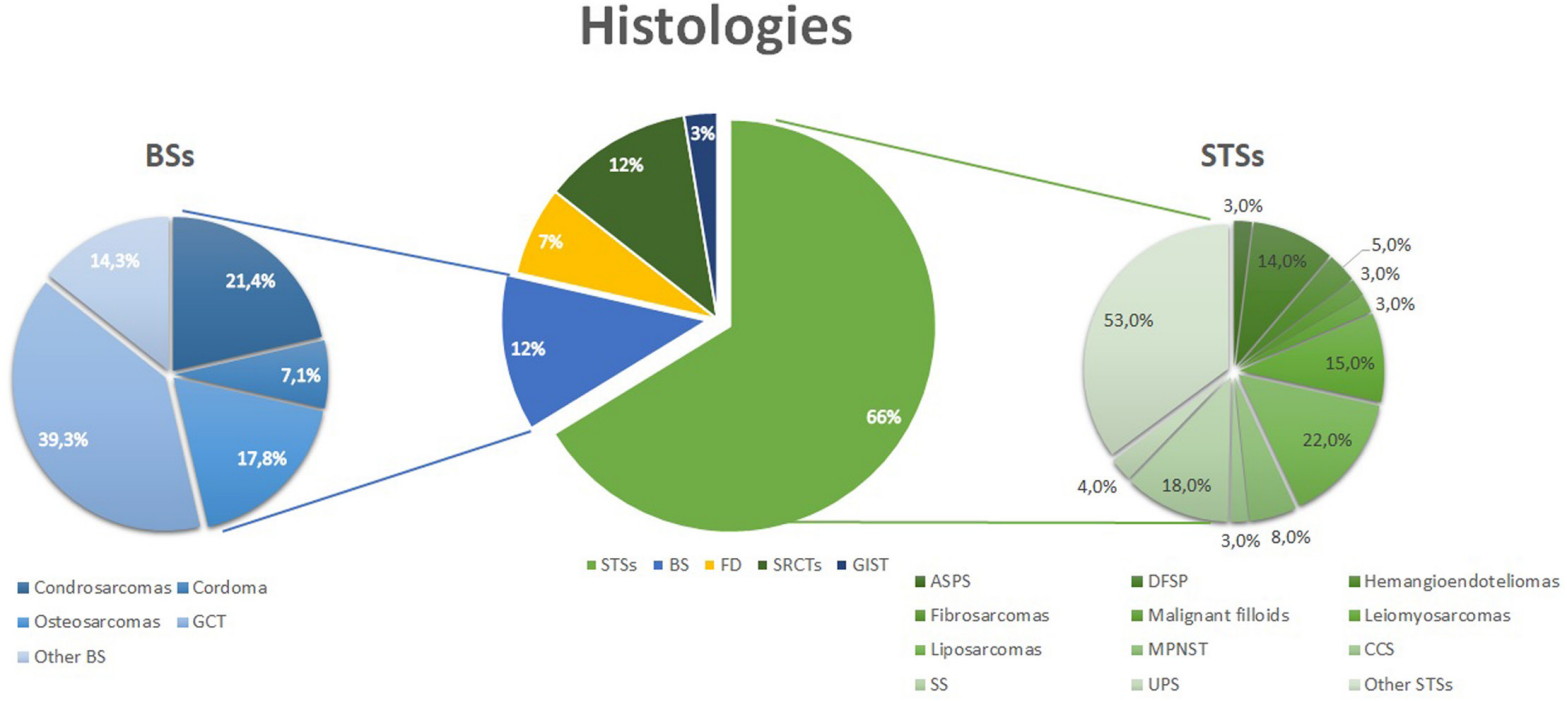
Different histologies among the sarcoma AYA patients. ASPS, alveolar soft part sarcoma; BSs, bone sarcomas; CCS, clear cells sarcoma; DFS, dermatofibrosarcoma protuberans; FD, desmoid fibromatosis; GCT, giant cell tumor; GIST, gastrointestinal stromal tumor; MPNST, malignant peripheral nerve sheath tumor; SRCTs, small round cells tumors; SS, synovial sarcoma; STSs, soft tissue sarcomas; UPS, undifferentiated pleomorphic sarcoma.

During the study period, we selected 228 patients aged 16–39 years with a diagnosis of soft tissue (STS) and bone sarcoma (BS) who were referred to our facility (Figure 1). The sample of whole AYA patients accounts for 277 among 2343 sarcoma patients (11.8% of the whole sarcoma population managed in our Institute), but we excluded 49 cases from the analysis due to the lack of follow‐up data. The median age was 30 years (ys) with 29% ≤ 25 years of age, 130 male (57%), and 98 (43%) female. Thirty‐five percent were smokers or former smokers (data available out of 188 patients). Out of 190 assessable patients, the median body mass index (BMI) at the diagnosis was 23.05 (range 12.42–46.81). We reported 7/228 (3%) cases of patients with a genetic syndrome: three cases with familial adenomatous polyposis (FAP), three cases with neurofibromatosis‐1, and 1 pt with Lynch syndrome.

Among 228 patients, 200 (88%) had STSs and 28 (12%) BS. Among STSs, 26/200 (13%) were small round cell tumors (SRCT) (21/200 extraskeletal Ewing's sarcomas, 3/200 embryonal and alveolar rhabdomyosarcomas, and 2/200 desmoplastic small round cell tumors, DSRCT). Among 103/200 (52%) intermediate–high‐grade, we identified 18/200 (9%) synovial sarcomas, 18/200 (9%) liposarcomas, 15/200 (7.5%) leiomyosarcomas, 9/200 (4.5%) angiosarcomas, 8/200 (4%) malignant peripheral nerve sheath tumors—MPNST, 3/200 (1.5%) clear cell sarcomas, 7/200 (3.5%) epithelioid sarcomas, and 3/200 (1.5%) alveolar soft part sarcomas—ASPS. Among 48/200 (24%) low‐grade STSs, we identified 14/200 (7%) dermatofibrosarcomas protuberans (DFSP) and 5/200 (2.5%) hemangioendotheliomas. Among BS (28/228, 12%), 9/28 (32%) were high‐grade (of which 4/28–14%—osteosarcomas and 3/28, 11%, chondrosarcomas) and 19/28 (68%) low‐grade (of which 11/28, 39%, giant cells tumors—GCT). Lastly, we also identified 16/228 (7%) patients with desmoid fibromatosis and 6/228 (2.6%) with gastrointestinal stromal tumor (GIST).

**FIGURE 2:**
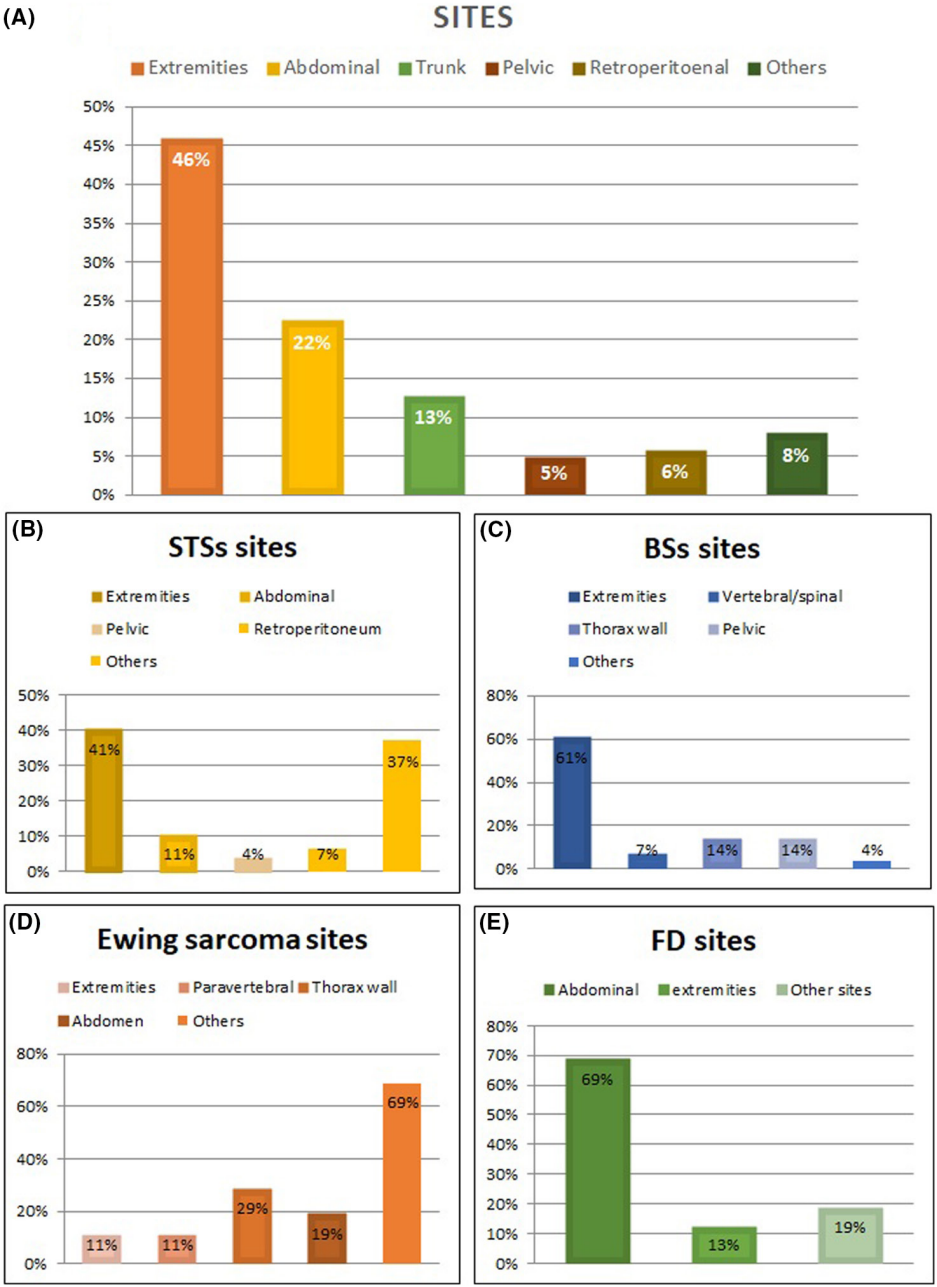
AYA patients described according to the site of sarcoma origin. (A) The whole study population according to the site of origin; (B) Soft tissue sarcomas, (C) Bone sarcomas, (D) Ewing's sarcomas, and (E) Desmoid fibromatosis by sites of origin.

Based on the tumor site, 105/228 (46%) patients presented with a primitive tumor originated in the extremities, 54/228 (23.7%) had primary abdominal disease, of which 13 in the retroperitoneum, 29/228 (12.7%) had trunk primary disease, and 12/228 (4.8%) had pelvic disease (Figure 2). The remaining 18 patients had different sites of presentation, less frequently involved. Among STSs, 81/200 (40.5%) originated from the extremities, 13/200 (6.5%) from the retroperitoneum, 21/200 (10.5%) from the abdomen, and 8/200 (4%) from the pelvis. Among BS, 17/28 (60.7%) patients presented with tumors localized in the extremities, 2/28 (7%) with vertebral/spinal disease, 4/28 (14%) originated from the thorax wall, and 4/28 (14%) from the pelvis. Ewing's sarcomas were extraskeletal, with the primary site in the thorax wall (so called Askin tumors) in 4/18 cases, paravertebral in 3/18 cases, pelvic in 2/18 cases, and in 3/18 from the extremities. Desmoid tumors were abdominal in 11/16 (69%) cases, while only three patients presented in the extremities or in the thorax wall.

**FIGURE 3:**
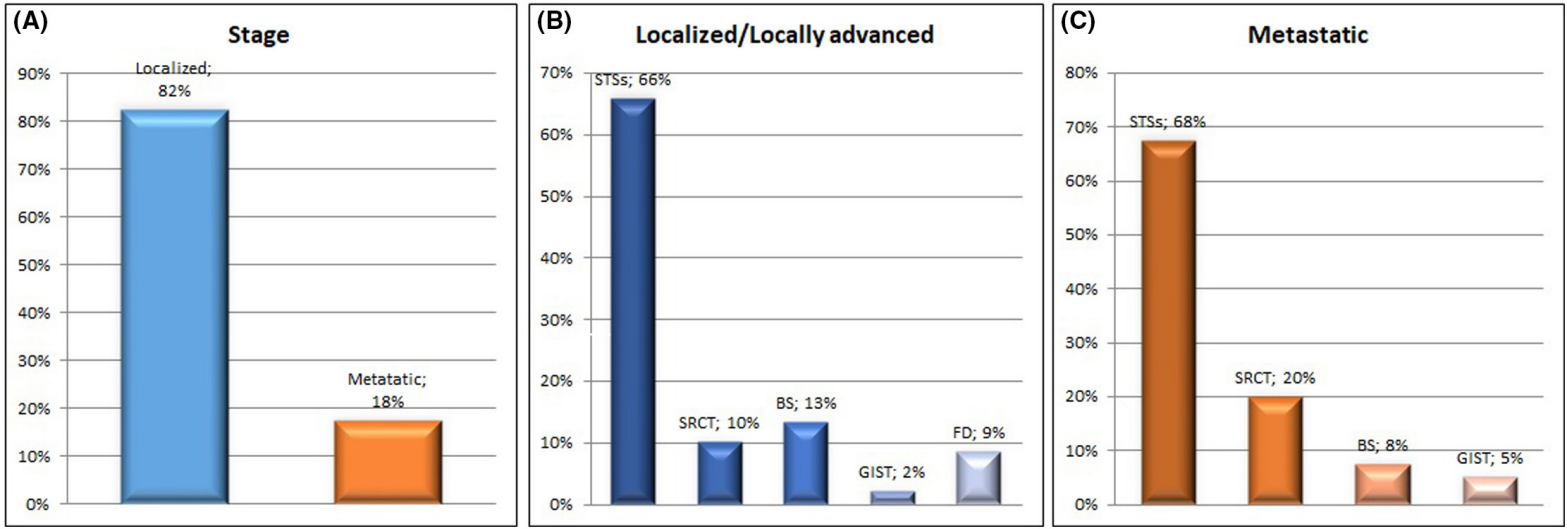
(A) AYA patients according to the sarcoma stage at diagnosis, (B) localized/locally advanced, and (C) metastatic.

Considering the extent of the disease, 188/228 (82.4%) patients presented a localized sarcoma at diagnosis, of which 80/188 (42.5%) with a primary tumor <5 cm of diameter. Among STSs, 124/200 (62%) of patients had localized disease, while among BS, only 3/28 (10.7%) were metastatic at diagnosis (Figure 3).

Among GIST, 2/6 (33%) patients had metastatic disease, while all desmoid fibromatosis were localized at diagnosis. Lastly, among Ewing Sarcomas, 16/21 (76%) had a localized/locally advanced disease at diagnosis.

**FIGURE 4:**
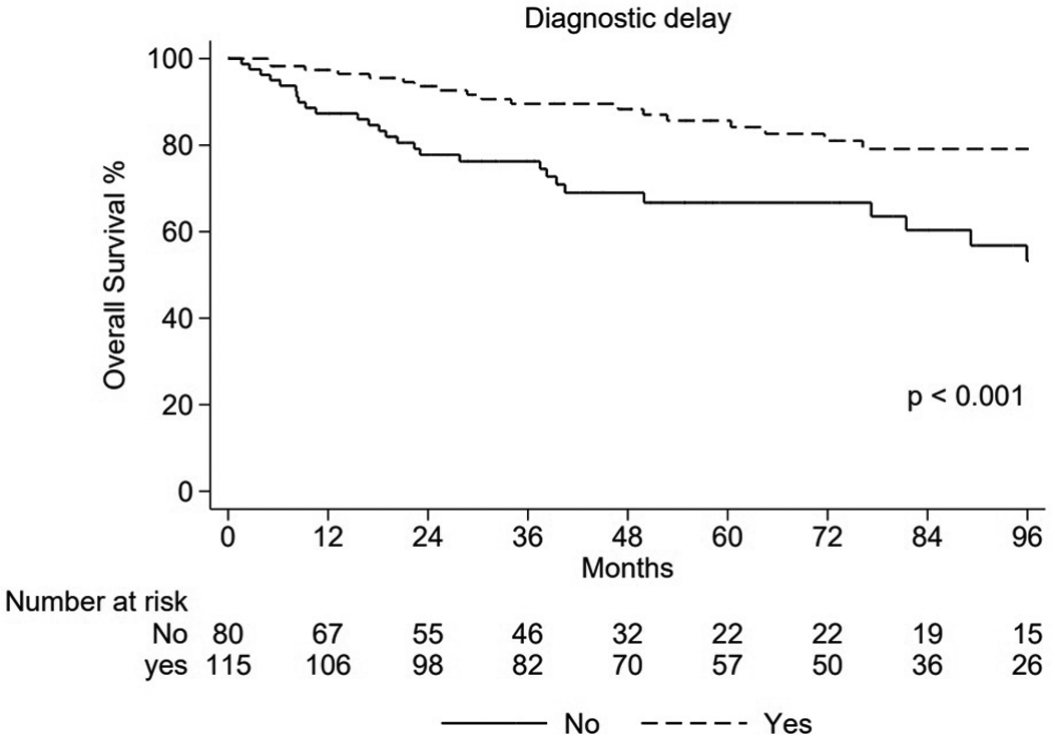
Overall survival stratified by time to diagnosis.

Out of 195/228 (86%) patients, the median TTD was 120 days (range 0–8255). The Kaplan–Meyer analysis showed a significantly better 5‐year OS for patients with >92 days of TTD (85.7% vs. 66.7%, *p =* 0.001) (Figure 4), also confirmed in intermediate–high‐grade sarcomas cohorts, separately analyzed.

We also reported a significantly better 5‐year PFS in intermediate–high‐grade sarcoma patients with a TTD > 92 days (50.2% vs. 24.9%, *p* = 0.009), but not for low‐grade (92.2% vs. 74.1%, *p =* 0.099). The population with a TTD < 92 days (80/195) was almost totally composed (51/80, 63.7%) by intermediate–high‐grade tumors, including small cells sarcomas.

### 3.1 Treatment of AYA patients

Overall, the therapeutic strategies included surgery, radiotherapy, systemic treatments and, in selected cases of SRCT, high dose chemotherapy, followed by peripheral blood stem cell infusion (PBSC). Among local treatments, surgery was performed in 190/228 (83%) patients, 185 with a curative intent on the primary tumor, and five for a progressive or relapsed disease, while radiotherapy in 99/228 (43.4%), 79 with a neo/adjuvant approach, and 20 for a palliative intent. Systemic therapies, including chemotherapy, target, and hormonal therapies, were performed in 150/228 (27%) patients, 124 as neo/adjuvant treatment according to a multidisciplinary approach, and 26 with a palliative intent for a relapse or progressive disease. Of all the patients receiving chemotherapy, 113/150 were treated with anthracyclines. Fourteen out of 228 (6%) SRCT patients underwent high dose chemotherapy, followed by PBSC, as consolidation therapy. The treatments of some peculiar histologic subgroups were reported as above. All Ewing's sarcoma patients received multiregimen systemic chemotherapy (including anthracycline, ifosfamide/cyclophosphamide, and vincristine), 14/21 (67%) underwent surgery, and 13/21 (62%) also received radiotherapy on the primary site. Based on the prognostic group (unfavorable axial site and high volume), 12/21 (57%) patients received the PBSC reinfusion after the conditioning regimen chemotherapy, including Busulfan and Melphalan. No toxic deaths were registered. Among desmoid tumors, 6/16 (37.5%) underwent surgery and 12/16 (68.75%) received systemic therapy: in particular, 11 received weekly chemotherapy with methotrexate/vinblastine and four hormonal treatment ± anti‐inflammatory drugs. Among GIST, 4/5 (80%) underwent surgery on the primary tumor and/or metastases, and 4/5 (67%) received systemic therapy with a tyrosine kinase inhibitor. Lastly, among GCT, 7/8 patients received systemic therapy with Denosumab, an effective RANKL inhibitor, three with a neo/adjuvant intent, and for local recurrence in the remaining four cases. The median TTT, available for the whole population, was 7 days (range 0–83), 75% of patients visited within 16 days. An increase of the risk of death with the increase of the number of treatments was observed (HR 1.36, CI 1.14–1.63, *p* < 0.001). Due to the heterogeneity of the whole sample, no outcome‐based analysis could be performed to evaluate the impact of each treatment strategy.

### 3.2 Outcome and follow‐up AYA

**FIGURE 5:**
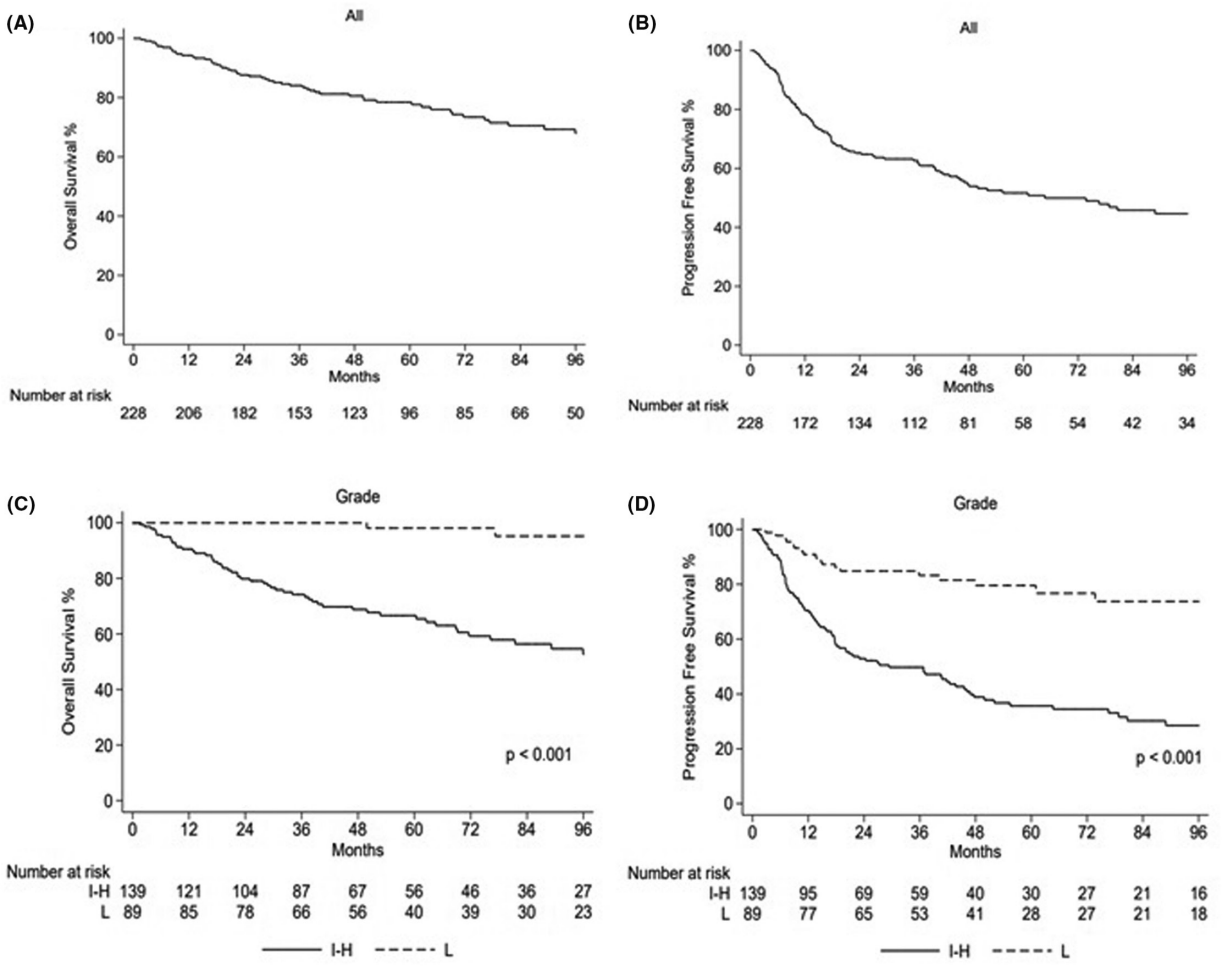
Trend of the overall survival (OS) and progression‐free survival (PFS). (A) OS in the whole study population; (B) PFS in the whole study population; (C) OS according to the sarcoma grade; (D) PFS according to the sarcoma grade. I‐H, intermediate/high‐grade sarcomas, L, low‐grade sarcomas.

**FIGURE 6:**
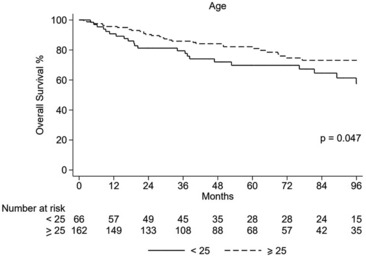
Overall survival in the study population according to age.

In the whole study population, with a median follow‐up from diagnosis of 72.9 months (range 1.6–145), 5‐year PFS and OS were 51.6% and 78.5%, respectively, and 10‐year PFS and OS were 42.9% and 62%, respectively (Figure 5). No impact on OS or PFS was reported for BMI and smoking. We reported a trend to a better OS for females versus males (5‐year OS 82.9% vs. 74.9%, *p* = 0.16), while no differences were underlined in terms of PFS. According to the grade, we reported a better 5‐year PFS for low‐grade versus intermediate–high‐grade sarcomas (79% vs. 36%, *p* < 0.001) and a better 5‐year PFS for localized versus metastatic disease (61% vs. 10%, respectively, *p* < 0.001). Kaplan–Meyer analysis reported a significant difference in 5‐year OS between intermediate–high‐risk versus low‐risk sarcomas (66.6% vs. 98.1%, *p* < 0.001), and a better prognosis for patients with localized versus metastatic disease (88.3% vs. 34% in 5‐year OS, *p* < 0.001). According to the sarcoma histotype, we reported a significantly different OS in 5‐year OS: we reported a 5‐year OS of 100% for all low‐grade STSs and desmoid fibromatosis, 75.9% for BS, 74.4% for intermediate–high‐grade STSs, 66.7% for GIST, and 45.6% for small cell sarcomas. Lastly, the 5‐year and 10‐year PFS for low‐grade histotypes were 79.4% e 68.7%, respectively, while 5‐year and 10‐year PFS for high‐grade were 35.6% e 28.5%, respectively (*p*: <0.001). According to the age cutoff of 25 years, 5‐year OS was 69.8% versus 82.2% for ≤25 years (68/228–29.8%) versus > 25 years (160/228–70.2%), respectively (*p* = 0.047), while the 5‐year PFS was 46.5% versus 53.9%, respectively (*p* value: 0.653). Excluding the SRCTs (most of them in the subgroup ≤25 years, 18/68 vs. 9/160) from this analysis, no differences were reported between the two subcategories (Figure 6). During the follow‐up period, 36/228 (15.7%) patients suffered from relapse, and 11/228 (4.8%) patients reported functional impairment due to local treatments (due to surgery and/or radiotherapy). Six out of 228 (2.6%) developed secondary tumors (two in a setting of a genetic syndrome, one breast cancer, one thyroid cancer, one Hodgkin lymphoma, and one urothelial cancer) and no clinically relevant anthracycline‐associated cardiac failure were reported.

## 4 DISCUSSION

The relevance of unmet clinical needs and the lack of outcome improvements in the last 20 years led the scientific community to give this category of nonpediatric/nonadult patients the dignity of a separate entity, worldwide recognized as AYA, and to promote dedicated programs/resources to this unique group of patients.

In our experience, even before the establishment of the AYA Center, we managed more than 200 AYA sarcoma patients, thanks to a multidisciplinary dedicated team including sarcoma and AYA experts, in order to satisfy the medical and psychosocial needs of young patients.

Our data do not confirm the assumption that diagnostic delay could be responsible for the worse prognosis in AYA patients. The diagnostic delay is historically mentioned as a negative factor in the prognosis of AYA cancers. The main reason for this delay seems to be the feeling of invincibility of AYA patients, which often leads to minimizing vague symptoms and avoiding an adequate checkup. In addition to this typical youthful attitude, the medical system plays a crucial role, having several reticences in suspecting cancer in such young patients.^8^, ^16^, ^17^, ^18^ Interestingly, in our analysis, we could confirm a relevant diagnostic delay (a mean of 120 days), but, despite the latter, the majority of patients (80%) received the diagnosis at the initial stage with localized disease. We did not demonstrate a negative correlation between TTD and prognosis, even in high‐grade sarcomas. The analysis was performed only on 195/228 patients. As information about the start of sarcoma‐related symptoms was lacking for 33 patients (14%). Actually, we found a worse outcome in patients with a shorter TTD (≤92 days). These unexpected findings overshadowed the impact of the well‐known diagnostic delay on AYA cancer's outcome, at least in a Referral Sarcoma Center as our Center, suggesting again that peculiar biological features and a high incidence of highly aggressive tumors, demanding a fast diagnostic assessment and therapeutic plan, are the predominant factors leading the prognosis.

Obviously, the significant positive role of a Referral Center led by a high level of multidisciplinary expertise has been proved to have a cost‐effectiveness impact on the management of rare cancers and could potentially impact the value of the diagnostic delay on survival.^19^, ^20^, ^21^, ^22^

We reported a significantly worse 5‐year OS in the youngest cohort (≤25 years old), which represents almost 30% of patients in our sample and harbors the highest incidence of SRCT, mostly extraskeletal Ewing's sarcomas and desmoplastic small round cell tumor. The prevalence of these histologies could explain the poorer OS, given that these tumors are typically diagnosed in pediatric patients, but in AYA patients are characterized by a worse clinical outcome, compared with pediatric patients.^16^, ^23^ We therefore confirm the poor prognosis in AYA population even when adopting pediatric‐oriented treatment regimens. Indeed, excluding SRCT, no difference in outcome was identified between age subgroups in the AYA population (≤25 or > 25 years old), overruling the impact of age and/or inappropriate treatment‐age related to the final outcome.

Our analysis confirmed previous published data not only about the epidemiology and clinical features, but also regarding the prognosis of sarcoma AYA patients,^24^, ^25^, ^26^ underlining the impact of a Referral Cancer Center with high expertise in the management of sarcoma as well as AYA patients care. In our sample, the distribution between soft tissue and bone sarcomas (88% and 12%, respectively) is coherent with an adult cancer center specialized in STSs as our Institute.

As recommended, every histological diagnosis of the present study was reviewed by a STS‐dedicated pathologist,^27^, ^28^, ^29^ the clinical history discussed in our MDT, guaranteeing the adherence to the international guidelines and offering a personalized therapeutic program.^30^ As per standardized recommendations, proper clinical management in some rare histotypes includes a “wait and see” approach (i.e., for desmoid tumors or some other low‐grade sarcomas).

Despite we did not demonstrate a significant role of smoking and overweight/obesity on sarcoma, they might represent risk factors for late effects (as second tumors, cardiovascular, and metabolic pathologies), directly or indirectly linked to clinical/psychosocial issues resulting from STS diagnosis and treatments.^31^, ^32^, ^33^ Hence, the importance of all actions aimed at promoting an appropriate management of late effects, from diagnosis until follow‐up (for example, anti‐smoking center, physical activity, and diet indications), in specific programs of survivorship.^8^, ^16^, ^17^, ^18^, ^34^

In conclusion, in our experience the youngest patients have the poorest prognosis, mainly due to the higher incidence of SRCT. Although AYA cancers belong to the category of rare cancer, and as such they suffer from a diagnostic and therapeutic delay/uncertainty, we did not confirm an association between TTD and poor outcome, presumably related to the variability of histology and clinical presentation. A centralized diagnosis and treatment in Referral Center appears mandatory to properly understand the unique and peculiar biology of cancer, facilitate a personalized approach, and improve the chances of accrual in clinical trials.^35^, ^36^ Therefore, the establishment of an AYA dedicated MDT in a Referral Center represents the logical development of such oncologic commitment as well as survivorship care.^37^ Moreover, a greater effort to promote cooperative multicentric studies may be sought to achieve a more comprehensive data collection and overcome obstacles to treatment care.

## AUTHOR CONTRIBUTIONS

**Alexia Bertuzzi:** Conceptualization (lead); data curation (lead); formal analysis (supporting); investigation (lead); methodology (lead); project administration (lead); resources (lead); supervision (lead); validation (lead); visualization (lead); writing – original draft (lead); writing – review and editing (lead). **Maria Susanna Grimaudo:** Conceptualization (supporting); data curation (supporting); formal analysis (supporting); investigation (supporting); methodology (supporting); resources (supporting); visualization (supporting); writing – original draft (supporting); writing – review and editing (supporting). **Alice Laffi:** Data curation (supporting); formal analysis (supporting); investigation (supporting); methodology (supporting); resources (supporting); validation (supporting); visualization (supporting); writing – original draft (supporting); writing – review and editing (supporting). **Laura Giordano:** Data curation (supporting); formal analysis (lead); writing – original draft (supporting); writing – review and editing (supporting). **Nicolò Gennaro:** Conceptualization (supporting); data curation (supporting); investigation (supporting); methodology (supporting); resources (supporting); visualization (supporting); writing – original draft (supporting); writing – review and editing (supporting). **Umberto Cariboni:** Conceptualization (supporting); resources (supporting); writing – original draft (supporting); writing – review and editing (supporting). **Licia Vanessa Siracusano:** Writing – original draft (supporting); writing – review and editing (supporting). **Vittorio Quagliuolo:** Data curation (supporting); resources (supporting); validation (supporting); writing – original draft (supporting); writing – review and editing (supporting). **Piergiuseppe Colombo:** Resources (supporting); writing – original draft (supporting); writing – review and editing (supporting). **D'Orazio Federico:** Resources (supporting); writing – original draft (supporting); writing – review and editing (supporting). **Salvatore Lorenzo Renne:** Resources (supporting); writing – original draft (supporting); writing – review and editing (supporting). **Cristina Specchia:** Writing – original draft (supporting); writing – review and editing (supporting). **Ferdinando Cananzi:** Resources (supporting); writing – original draft (supporting); writing – review and editing (supporting). **ANDREA MARRARI:** Resources (supporting); writing – original draft (supporting); writing – review and editing (supporting). **Pierina Navarria:** Resources (supporting); writing – original draft (supporting); writing – review and editing (supporting). **primo daolio:** Resources (supporting); writing – original draft (supporting); writing – review and editing (supporting). **stefano bastoni:** Resources (supporting); writing – original draft (supporting); writing – review and editing (supporting). **Armando Santoro:** Supervision (supporting); visualization (supporting); writing – original draft (supporting); writing – review and editing (supporting).

## FUNDING INFORMATION

This work was funded by BIBLIOSAN.

## CONFLICT OF INTEREST STATEMENT

The authors declare no conflict of interest.

## Data Availability

The data that support the findings of this study are available from the corresponding author upon reasonable request.
